# Pawedness Trait Test (PaTRaT)—A New Paradigm to Evaluate Paw Preference and Dexterity in Rats

**DOI:** 10.3389/fnbeh.2017.00192

**Published:** 2017-10-16

**Authors:** Ana M. Cunha, Madalena Esteves, Sofia P. das Neves, Sónia Borges, Marco R. Guimarães, Nuno Sousa, Armando Almeida, Hugo Leite-Almeida

**Affiliations:** ^1^Life and Health Sciences Research Institute (ICVS), School of Medicine, University of Minho, Braga, Portugal; ^2^ICVS/3B’s—PT Government Associate Laboratory, Braga, Portugal

**Keywords:** motor preference, laterality, handedness, cognition, impulsivity, memory, behavior

## Abstract

In rodents, dexterity is commonly analyzed in preference paradigms in which animals are given the chance to use either the left or the right front paws to manipulate food. However, paw preference and dexterity at population and individual levels are controversial as results are incongruent across paradigms. We have therefore developed a semi-quantitative method—the **pa**wdeness **tra**it **t**est (PaTRaT)—to evaluate paw preference degree in rats. The PaTRaT consists in a classification system, ranging from +4 to −4 where increasingly positive and negative values reflect the bias for left or right paw use, respectively. Sprague-Dawley male rats were confined into a metal rectangular mesh cylinder, from which they can see, smell and reach sugared rewards with their paws. Due to its size, the reward could only cross the mesh if aligned with its diagonal, imposing additional coordination. Animals were allowed to retrieve 10 rewards per session in a total of four sessions while their behavior was recorded. PaTRaT was repeated 4 and 8 weeks after the first evaluation. To exclude potential bias, rats were also tested for paw fine movement and general locomotion in other behavioral paradigms as well as impulsivity (variable delay-to-signal, VDS), memory and cognitive flexibility (water maze). At the population level 54% of the animals presented a rightward bias. Individually, all animals presented marked side-preferences, >2 and <−2 for left- and right-sided bias, respectively, and this preference was stable across the three evaluations. Inter-rater consistency was very high between two experienced raters and substantial when two additional inexperienced raters were included. Left- and right-biased animals presented no differences in the ability to perform fine movements with any of the forelimbs (staircase) and general locomotor performance. Additionally, these groups performed similarly in executive function and memory tasks. In conclusion, PaTRaT is able to reliably classify rats’ pawedness direction and degree.

## Introduction

Pawedness reflects the preferential use and/or an increased capacity to perform tasks more efficiently with a specific paw. It corresponds, in general terms, to animals’ handedness. Pawedness/handedness is thought to be associated with brain asymmetries, present both at morphological, cellular and molecular levels (see for reviews Galaburda et al., 1978; Toga and Thompson, 2003; Sun and Walsh, 2006; Rogers, 2009, 2014; Hugdahl, 2011). Regarding morphology most studies have so far excluded any association (Good et al., 2001; Narr et al., 2007; Guadalupe et al., 2014, 2016; Ocklenburg et al., 2016); however at cellular and molecular levels, contralateral parietal spine density has been linked to skilled reaching (Ambeskovic et al., 2017) and dopaminergic system lateralization has been shown to be associated with hand/paw preference in humans (de la Fuente-Fernández et al., 2000) as well as in rodents (Uguru-Okorie and Arbuthnott, 1981; Schwarting et al., 1987; Barnéoud et al., 1990; Cabib et al., 1995; Nielsen et al., 1997; Budilin et al., 2008). Additionally, peripheral human (Lengen et al., 2009) and central rodent (Neveu, 1990; Shen et al., 2005) data have shown differences in the immunological system, while additional monoamines (norepinephrine; Barnéoud et al., 1990) and enzymes (angiotensinases; Wu et al., 2010) were also associated with rodent pawedness. It has therefore been hypothesized that differences in this trait might be associated with other behavioral outcomes particularly cognition. Indeed, a small advantage of right-handed people in spatial ability has been reported (Somers et al., 2015) and pawedness/memory associations have been found in monkeys (Hopkins and Washburn, 1994) and mice (Wu et al., 2010). Furthermore, Prichard et al. (2013) reported that cognitive data is associated with the degree of handedness (and not its direction) as inconsistent handedness seems to be related with better episodic memory and improved belief updating/cognitive flexibility.

While handedness assessment in humans is simple, determination of associated behavioral and molecular differences poses several challenges: (i) the distribution of left- and right-handers in the population is uneven (Teng et al., 1976; Guadalupe et al., 2016) imposing the creation of specific left-enriched cohorts; (ii) social context may alter behavior (Teng et al., 1976), therefore increasing the percentage of strong right-handers and weak left-handers; and (iii) assessment of central molecular correlates is limited. The utilization of animal models became therefore very useful in this regard.

The Collins’ (Collins, 1968) and the lateral paw preference tests (LPP; Waters and Denenberg, 1991) are amongst the most used tests to assess pawedness in rodents. The number of times a paw is used to retrieve food from an elevated tube and the amount of food retrieved from two lowered hoppers placed side-by-side are employed, respectively, as behavioral readouts. Despite the construct similarities, different results have been obtained between these two tests (Waters and Denenberg, 1991, 1994; Rogers and Bulman-Fleming, 1998) not only at the population level but, more importantly, at the individual level. Furthermore, both tests rely in the exclusive right/left paw use disregarding paw movement precision and possible intermediary strategies implicating the simultaneous use of both paws. Additionally, as measured by these methods, preference in rodents appears to a certain extant to be training/learning dependent (Collins, 1988; Stashkevich and Kulikov, 2001; Tang and Verstynen, 2002; Ribeiro et al., 2011).

To surpass these limitations, we have designed and validated an alternative test requiring minimal equipment for a fast determination of pawedness degree—the **pa**wdeness **tra**it **t**est (PaTRaT). In this test, a circular grid separates the animal from a receptacle containing sugared items. Contrary to previous tests, the PaTRaT allows the simultaneous use of the two paws for reward handling reducing potential selection biases and making it more ethologically relevant. Also, the large size of the reward compared to the grid slits, imposes higher movement complexity for successful retrieval. Finally, the PaTRaT uses a classification system for dexterity degree that goes beyond the simple quantification of left-/right-paw retrievals. Additionally, we assessed potential associations between pawedness and behavioral outcomes, namely with impulsivity, memory and fine motor skills.

## Materials and Methods

### Animals

Thirty male Sprague-Dawley rats (Charles-River Laboratories) with 6 months of age were kept under standard laboratory conditions: 12 h light/dark cycle (lights on at 8 a.m.), relative humidity of 55%, 22°C and *ad libitum* access to water. Food (4RF21, Mucedola SRL) was restricted to 1 h per day (last hour of the cycle light phase) during experimental protocols otherwise access was *ad libitum*. Body weight was controlled on a weekly basis to prevent weight losses superior to 15%. Animals that failed to learn the PaTRaT in the training sessions (see below) were excluded from further analysis. Procedures involving animals were approved by local authorities and followed the EU Directive 2010/63/EU.

### Pawedness Trait Test (PaTRaT)

#### Apparatus

**Figure 1:**
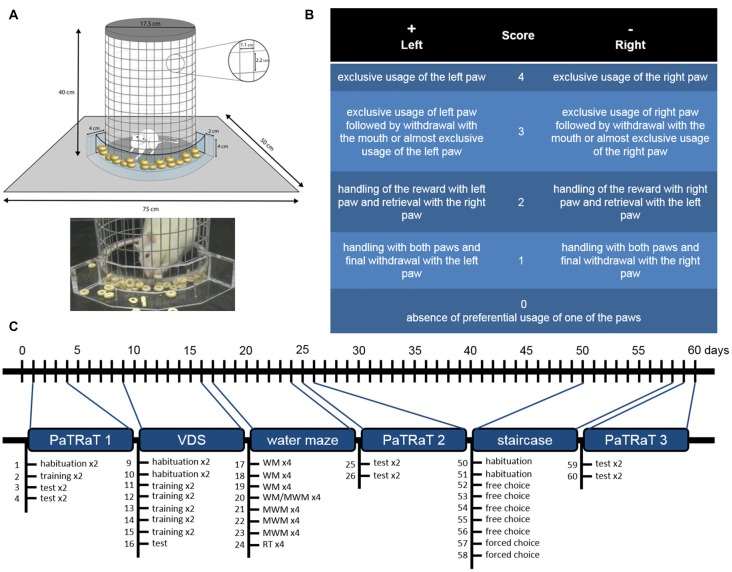
Methods. **(A)** Scheme (top) and picture (bottom) depicting the pawedness apparatus. The animal is enclosed in a gridded cylinder. An exterior plexiglass structure contains the rewards. **(B)** Schematic table of classification for each retrieved reward. Positive and negative values correspond respectively to preferential usage of left and right paw and higher values are associated to more exclusive usage of said paw. **(C)** Timeline of behavioral analysis. Pawedness was assessed three times approximately 4 weeks apart. VDS and water mazes were performed between the two first evaluations and staircase test was performed before the third pawedness evaluation. Individual sessions are specified. VDS, Variable Delay-to-Signal; WM, working memory; MWM, Morris Water Maze; RT, reversal test.

The PaTRaT apparatus consisted of a custom-made plexiglass box open at the top. In the center, a metal wire mesh cylinder was used to confine the animal. Externally, accompanying cylinder’s curvature, a plexiglass transparent piece was fixed to the bottom forming a receptacle for the rewards (Figure 1A).

#### Experimental Protocol

##### Training

Two daily sessions were performed, separated by a minimum of 4 h. Animals were habituated to the apparatus (1 session, 10 min) and to the reward (Cheerios^®^, Nestlé; 1 session, 10 min). On the next sessions, animals were motivated to reach for the reward with their paws by placing single rewards close to the grid (1–2 sessions, 15 min). The diameter of the reward was larger than the horizontal width of the metal mesh, increasing the demanding for successful retrievals.

##### PaTRaT

Four experimental sessions were performed, in which the animal had to retrieve 10 rewards in a maximum time of 10 min while the session was being recorded. Sessions were performed during the light period (8:00–12:00 and 14:00–18:00 for morning and afternoon sessions, respectively). Between animals the apparatus was cleaned with 10% ethanol.

To evaluate phenotype stability, two additional evaluations were performed 4 and 8 weeks after the first evaluation. Sessions were recorded and later evaluated independently by two observers. Additionally, the first evaluation was also rated by two inexperienced observers to assess inter-rater reliability.

##### Behavioral rating

Paw dexterity was determined by averaging the 40 trials (i.e., reward retrievals) of each evaluation. Each successful reward withdrawal was classified in a scale of +4 to −4. Positive and negative values reflected preferential use of the left or right paw respectively and increasing values were associated with increasing preference. Classification was only attributed when a reward was retrieved; unsuccessful attempts were not rated. Rewards withdrawn without usage of any of the paws (i.e., with the mouth) were also not classified.

Score 4 corresponded to exclusive usage of the left paw; score 3 to exclusive usage of left paw followed by withdrawal with the mouth or to almost exclusive usage of the left paw (e.g., right paw used as support); score 2 was attributed when the animal handled the reward with its left paw but retrieved it with the right paw; and score 1 corresponded to handling with both paws and final withdrawal with the left paw. Score 0 was associated with absence of preferential usage of one of the paws, namely equal usage of both paws for reaching the reward and final withdrawal with the mouth. Symmetrical values corresponded to similar classification for the opposite paw (Figure 1B).

### Additional Behavioral Tests

In order to assess potential lateral preference-behavior associations, other behaviors were assessed (Figure 1C).

#### Variable Delay-to-Signal Test (VDS)

The variable delay-to-signal (VDS) task was used to assess impulsive behavior. It was previously validated by drug-induced changes in impulsivity and by comparison with reference paradigms (Leite-Almeida et al., 2013). In short, the test was performed in a 5-hole operant chamber in which only the middle nosepoke aperture was available. Animals performed two daily sessions separated by a minimum of 5 h for a total of 8 days. There were four habituation sessions: the first two had the duration of 15 min, lights were off, all nosepoke apertures were blocked and sugared pellets (45 mg, Bioserv Inc., Flemington, NJ, USA) were available at the food magazine. The latter two had the duration of 30 min, all lights were on and pellets were available at both the food magazine and the nosepoke aperture.

Training consists of 10 sessions of 100 trials (or 30 min) in which there was a 3 s’ interval between the beginning of the trial and the lightning of the nosepoke aperture (delay period). If a nosepoke was performed in this period, it was considered a premature response and it was punished with a 5 s’ timeout in complete darkness. If the nosepoke was performed in the 60 s’ period in which the light in the aperture was on, it was considered as a correct response and a reward was retrieved in the food magazine, initiating a new trial. If no nosepoke was performed, it was considered an omission and the animal was punished with a timeout.

Testing consisted of a single session at the end of the training. It was constituted by a total of 120 trials (or 90 min), in which premature responses were not punished and the intervals were variable. It started with 25 3 s’ trials (3si), followed by 70 6 s (6 s) and 12 seconds’ (12 s) (randomized) trials and again by 25 3 s’ trials (3sf). During the testing session multiple premature responses were allowed during the delay periods and the rate of premature responses per time of available delay was calculated (Leite-Almeida et al., 2013).

#### Water Maze Test

In order to test memory, animals were subjected to a modification of the Morris Water Maze (MWM) test (Morris, 1984), for which the procedures have been previously described (Cerqueira et al., 2007). Briefly, animals were placed in a black tank 170 cm in diameter filled with 31 cm of water at 22°C. The tank was divided in four virtual quadrants, each associated with an external visual clue. A non-visible platform (black, 12 cm diameter, 30 cm high) was placed inside the tank and all movements were recorded through a video camera on the ceiling and tracked using a video-tracking system (Viewpoint, Champagne au mont d’or, France). In all trials the animal had 120 s to find the platform, at the end of which it was gently pushed towards if unable to complete the task. After reaching the platform, the animal was allowed 20 s on it before starting a new session.

Evaluation of working memory (WM) consisted of 4 days of evaluation, four sessions each. The position of the platform was maintained within each day, but changed on consecutive days, while the animal initiated each session on a different quadrant. The last day of WM evaluation was also the first of 4 days of MWM testing, in which the platform remained on the same place throughout all days of testing, while all remaining parameters were similar to the WM test. On the final day of testing, a reversal test (RT) was performed. Here, the platform (which had been in the same quadrant for 4 days) was moved to the opposite quadrant and four sessions equal to the ones above described were performed.

In all modalities, time to reach platform was evaluated. For WM and MWM, the average evolution curves throughout first to fourth trial or day were assessed respectively. For RT, the comparison was between time spent in the new and old quadrants.

#### Staircase Test

Aiming to assess potential differences in motor skills between left and right pawed animals, a modified version of the original staircase test (Montoya et al., 1991) was performed, for which most procedures have been previously described (Teixeira et al., 2017). This test required the usage of double staircase boxes (Campden Instruments, Lafayette, IN, USA), which consists of a narrow platform connected to a larger chamber with a moveable lid. A double removable seven-step staircase was inserted along both sides of the platform. In each session, five sugared pellets (45 mg, Bioserv Inc., Flemington, NJ, USA, EUA) were placed in each step and one daily session was performed. The first two sessions aim to habituate the animals and last respectively 5 and 10 min each, after which five test sessions were performed, each lasting 5 min. The last two evaluations were forced choice sessions, in which only one of the staircases (left or right) had pellets in it. In all cases, at the end of the session, the remaining pellets were counted.

Measures of interest were reached level at each side (lowest level from which pellets were withdrawn) and success rate (number of pellets eaten/total number of pellets) in both normal and forced sessions.

### Statistical Analysis

All statistical analyses were performed on Matlab R2009b software. *P* < 0.05 was always considered the significance threshold. For assessment of inter-rater reliability Kappa statistics was used, namely Fleiss’ Kappa when comparing between four observers and Cohen’s Kappa when comparing the two experienced raters. Potential time-dependent differences in pawedness were assessed by comparing three separate time points using a repeated measures ANOVA.

For analysis of behavior mixed design ANOVAs were conducted. For VDS, the between subjects factor was pawedness group and the within subjects factor was session (training) or interval (test). For water maze, the between subjects factor was also pawedness group and the within subjects factor was either trial (WM), day (MWM) or quadrant (RT).

Differences in motor performance and motivation were assessed using simple group comparison. As normality could not be confirmed, non-parametric tests were used.

## Results

### Pawedness (Inter-Rater Agreement)

**Figure 2:**
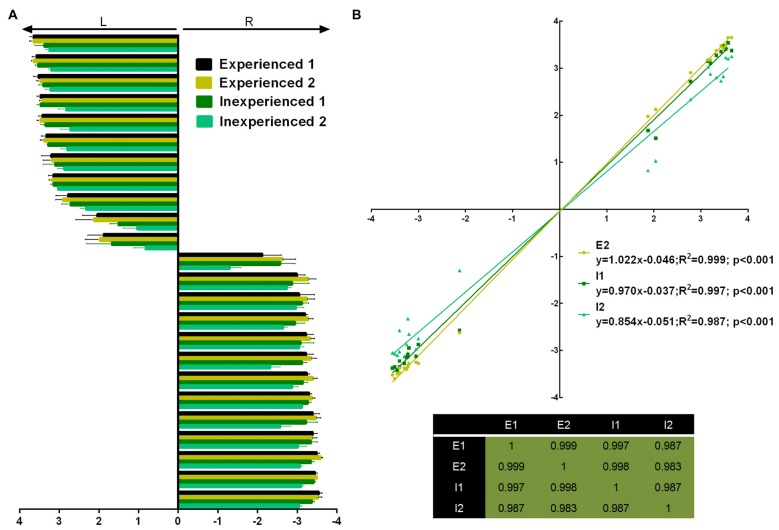
Pawdeness trait test (PaTRaT) rater comparison. Two experienced and two inexperienced raters scored the first pawedness evaluation. **(A)** Representation of the score attributed by each rater for each animal shown as mean ± SEM. **(B)** Experienced rater 1’s scores correlated with scores attributed by all other raters (graph) and all raters show high correlation between them (table of *R*^2^s). L, Left; R, Right; E, experienced; I, inexperienced.

Four observers (two experienced and two inexperienced) rated the first pawedness evaluation (Figure 2A). Inter-rater agreement was assessed and rendered a “substantial agreement” (Fleiss’ Kappa = 0.615; *p* < 0.001; 95% CI = 0.590–0.640). Additionally, individual raters’ scores showed to be linearly correlated among them (Figure 2B).

### Pawedness (Temporal Stability)

**Figure 3:**
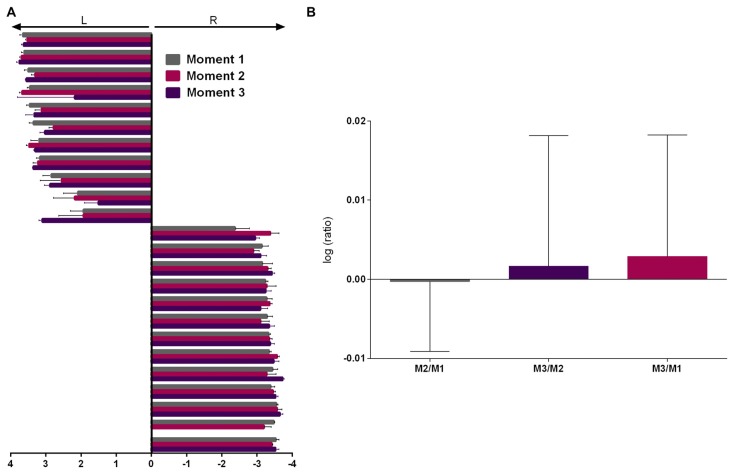
PaTRaT time-related stability. Pawedness was evaluated at three separate moments. **(A)** Representation of the score attributed at each moment. 11/13 animals showed left/right preference, respectively. **(B)** Graph shows the logarithmic ratios between the three moments of evaluation. Data is shown as mean ± SEM. L, Left; R, Right; M, moment.

As the two experienced raters presented very high correlation and inter-rater agreement (Cohen’s kappa = 0.932, *p* < 0.001, 95% CI = 0.762–1.102) on the first evaluation, this data was averaged between them and subsequent evaluations were assessed by both (2 sessions each) and averaged. Repeated measures ANOVA showed no effect of moment (M) of evaluation (Figure 3A, *F*_(2,44)_ = 0.641, *p* = 0.532) and analysis of the logarithmic ratio between moments of evaluation showed no difference from 0 (Figure 3B, M2/M1: *Z* = 0.400, *r* = 0.082, *p* = 0.689; M3/M2: *Z* = 1.156, *r* = 0.241, *p* = 0.248; M3/M1: *Z* = 1.247, *r* = 0.260, *p* = 0.212).

Thus, from the total of 24 evaluated animals, all showed consistent paw preference across time. From these, 11 and 13, respectively showed left and right paw preference. Following veterinary decision one animal was excluded prior to M3. Up to this timepoint general aspects of well-being, weight and other behavioral measures were normal.

### Variable Delay-to-Signal (Impulsivity)

**Figure 4:**
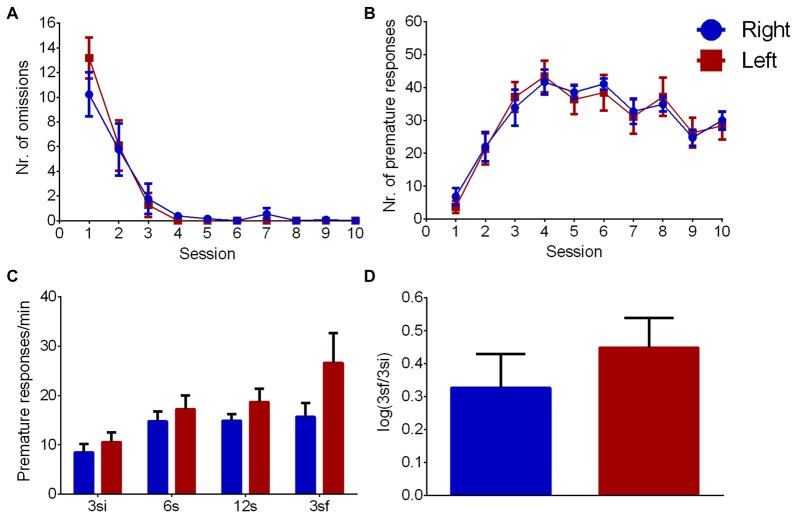
Left/Right differences in impulsivity. Group comparison during VDS training **(A,B)** and test **(C,D)**. **(A)** Number of omissions, **(B)** number of premature responses, **(C)** premature responses per minute per interval and **(D)** logarithmic ratio of the number of premature responses in 3sf and 3si intervals. Data is shown as mean ± SEM. Blue—animals with right paw preference; Red—animals with left paw preference.

During the learning phase, the evolution number of omissions across sessions (Figure 4A) showed an effect of session (*F*_(9,198)_ = 40.428, *p* < 0.001), but no effect of pawedness group (*F*_(1,22)_ = 0.060, *p* = 0.809) or interaction session/group (*F*_(9,198)_ = 0.690, *p* = 0.718) indicating that both groups learned equally well the task. Similar results were found for correct nosepokes (session: *F*_(9,198)_ = 20.377, *p* < 0.001; group: *F*_(1,22)_ = 0.007, *p* = 0.935; interaction: *F*_(9,198)_ = 0.529, *p* = 0.852) and premature responses (Figure 4B—session: *F*_(9,198)_ = 38.350, *p* < 0.001; group: *F*_(1,22)_ = 0.005, *p* = 0.943; interaction: *F*_(9,198)_ = 0.415, *p* = 0.926). Left and right pawed animals thus showed no differences in learning the task.

Regarding impulsivity (Figure 4C), which is evaluated by delay intolerance (premature responses per min), there was a significant effect of interval (*F*_(3,66)_ = 12.617, *p* < 0.001) but no effect of group (*F*_(1,22)_ = 2.095, *p* = 0.162) or interaction (*F*_(3,66)_ = 2.371, *p* = 0.078). Similarly, log (3sf/3si) showed no differences between animals with left and right paw preference (Figure 4D, *Z* = 0.758 Cohen’s *d* = 0.377, *p* = 0.448).

### Water Maze (Memory)

**Figure 5:**
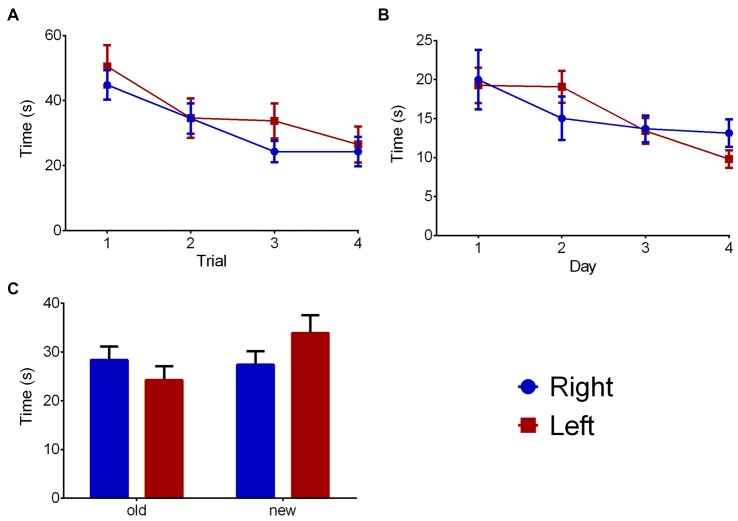
Group effects on memory. Group comparison on the water maze test in WM **(A)**, classic Morris **(B)** and reversal **(C)** components. Data is shown as mean ± SEM. Blue—animals with right paw preference; Red—animals with left paw preference.

The WM part of the water maze (Figure 5A) trial showed a significant effect on time to reach the platform (*F*_(3,66)_ = 8.487, *p* < 0.001), but no influences of pawedness group (*F*_(1,22)_ = 1.104, *p* = 0.305) or interaction (*F*_(3,66)_ = 0.373, *p* = 0.773) were found. Similar data was found regarding MWM (Figure 5B—day: *F*_(3,66)_ = 1.075, *p* = 0.001; group: *F*_(1,22)_ = 0.001, *p* = 0.974; interaction: *F*_(3,66)_ = 1.075, *p* = 0.366). No effects were found on the RT component of the test (Figure 5C—quadrant: *F*_(1,22)_ = 1.291, *p* = 0.268; group: *F*_(1,22)_ = 0.387, *p* = 0.540; interaction: *F*_(1,22)_ = 1.932, *p* = 0.178). In all cases, similar results were found when analyzing distance traveled rather than time to reach the platform (data not shown).

### Fine Motor and Locomotor Performance

**Figure 6:**
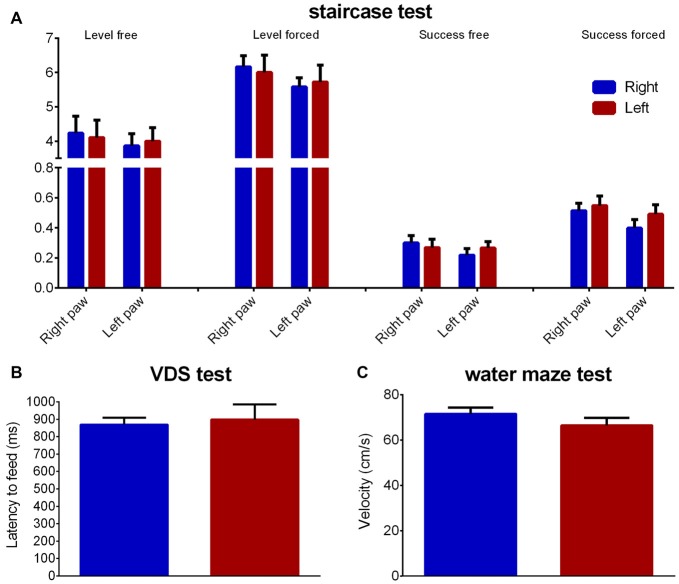
Fine motor and locomotor performance. Group comparison regarding fine motor coordination (**A**—staircase test), motivation to eat (**B**—latency to feed in the VDS test) and motor performance (**C**—velocity in the water maze). Data is shown as mean ± SEM. VDS, Variable Delay-to-Signal; Blue—animals with right paw preference; Red—animals with left paw preference.

The staircase test showed no group differences regarding fine motor coordination for both right (level normal: *Z* = 0.434 Cohen’s *d* = 0.105, *p* = 0.664; level forced: *Z* = 1.074 Cohen’s *d* = 0.110, *p* = 0.283; success rate normal: *Z* = 0.838 Cohen’s *d* = 0.334, *p* = 0.402; success rate forced: *Z* = 1.131 Cohen’s *d* = 0.455, *p* = 0.258) or left (level normal: *Z* = 0.203 Cohen’s *d* = 0.073, *p* = 0.839; level forced: *Z* = 0.602 Cohen’s *d* = 0.117, *p* = 0.548; success rate normal: *Z* = 0.260 Cohen’s *d* = 0.177, *p* = 0.795; success rate forced: *Z* = 1.389 Cohen’s *d* = 0.176, *p* = 0.165) paws (Figure 6A).

Regarding latency to reward retrieval in the VDS no differences between animals with left or right paw preference were found (*Z* = 0.513 Cohen’s *d* = 0.124, *p* = 0.608), as seen through the time to retrieve the reward during the VDS test (Figure 6B). Additionally, no differences were found in the water maze average swimming velocity (*Z* = 0.051 Cohen’s *d* = 0.486, *p* = 0.959; Figure 6C).

## Discussion

PaTRaT is a grading system to evaluate pawedness in rats. It is of simple implementation, requiring minimal equipment. Also, it has high inter-rater reliability and temporal stability of the outcomes. The PaTRaT displays several advantages over the most used tests—the Collins’ (Collins, 1968) and LPP (Waters and Denenberg, 1991) tests, namely: (i) the reward can be readily seen, smelled and touched by the animals, increasing motivation. Indeed, we observed in preliminary assays that animals were more prone to perform the test when a greater amount of reward (≈50 vs. ≈20 cheerios^®^) was available (data not shown); (ii) the apparatus imposes no constraints to the simultaneous use of both paws for reward manipulation, decreasing potential selection biases (Ribeiro et al., 2011) and providing a more ethological setting; (iii) the large reward size relative to the mesh grid imposes higher dexterity, i.e., a sequence of relatively complex movements is required for a successful retrieval of the reward; (iv) assessment is based on four independent sessions (10 trials each) avoiding potential confounders as limb alternation due to tiredness and satiation (Bulman-Fleming et al., 1997); (v) the grading system is of simple application and has a high inter-rater agreement even among inexperienced observers; and finally (vi) PaTRaT side preference index, contrary to the simple quantification of left/right paw retrievals, accounts for intermediate pawedness and is therefore more akin to human handedness assessment (Prichard et al., 2013). Additionally, two sessions were enough to achieve a sustained performance in 80% of the animals. In our study, the prevalence of right- and left-pawed rats was similar (54% vs. 46%, respectively), which is in accordance with a previous report (Stashkevich and Kulikov, 2001) but in opposition with others that report a rightward bias up to ≈82.4% (Pençe, 2002; Elalmis et al., 2003; Güven et al., 2003; Wu et al., 2010). Scores were stable across the three evaluations even when other manipulations/behavioral paradigms were interposed; potential differences in fine motricity (or even in general motor performance) were experimentally excluded. Importantly, we evaluated the PaTRaT in male Sprague-Dawley rats but strain, sex and species differences have been described in the context of paw preference protocols (see for example Betancur et al., 1991).

It has been argued that a less marked lateralization in rodents (or other animals) in comparison with humans results from the fact that pawedness assessments rely on the evaluation of simple movements (e.g., grabbing food) instead of fine movements as in humans (see for review on nonhuman primates, Hopkins, 2013). Thus, the increased movement complexity of the PaTRaT assay may explain the absence of ambidextrous animals, normally reported as being 7.4%–23% (Stashkevich and Kulikov, 2001; Pençe, 2002; Elalmis et al., 2003; Güven et al., 2003; Wu et al., 2010), as it allows a better separation of left- and right-pawed animals (i.e., average scores close to the limits of the scale). In fact, decreases in the PaTRaT absolute scores were mostly related with poorer performances of the preferred paw and not with retrievals with the non-preferred paw (practically absent).

Pawedness in rats has been associated with central asymmetries in monoamines, notably dopamine and other molecular players (Barnéoud et al., 1990; Cabib et al., 1995; Budilin et al., 2008). These have been hypothesized to underlie associations between pawedness direction (and/or magnitude) and performance in several behavioral domains (Wu et al., 2010)—see also Prichard et al. (2013) for a comprehensive review in human studies. Specifically regarding impulsive behavior, several studies have demonstrated an influence of the dopamine levels (see for review, Dalley and Roiser, 2012; D’Amour-Horvat and Leyton, 2014). We have nevertheless observed no differences between left- and right-biased animals on impulsive behavior both on the learning protocol or in the VDS test. Additionally, no differences were observed in long-term and working spatial reference memories and reversal learning.

In conclusion, the PaTRaT is a simple, inexpensive and reliable test for assessment of pawedness degree and direction in rats. It relies on a grading system for hind paw use and has high inter-rater agreement (even for inexperienced observers). At the population level, we observed a nearly equal distribution of left- and right-biased rats and individual preferences were stable across sessions.

## Author Contributions

AMC, ME and HL-A designed the experiment; AMC, ME and MRG performed the experiments, AMC, ME, SPN and SB rated the animals; ME and HL-A performed the statistical analysis; AMC, ME, NS, AA and HL-A analyzed and interpreted the data; AMC, ME and HL-A wrote the article’s initial version. All authors revised and approved the final version of the manuscript.

## Conflict of Interest Statement

The authors declare that the research was conducted in the absence of any commercial or financial relationships that could be construed as a potential conflict of interest.
